# Dr. Charles S. Stockton

**Published:** 1912-10-15

**Authors:** 


Dr. Charles S. Stockton.—Dr. Stockton was the best known dentist of New Jersey both at home and throughout the country. He began practicing in New Jersey in 1857, without a college degree; but attended the Pennsylvania College of Dental Surgery and took his degree in 1868. He was born in New Jersey in 1836 and died at his home in Newark, N. J., September 9, 1912. He was intensely interested in his profession all his life and gave generously of his time and money for the promotion of every effort made for the advancement of dentistry to larger and better attainments both at home and nationally. He was a charter member of the State Society and took an active part in promoting every local and state organization in his profession. One of his last activities was the promotion of the Newark free dental clinic, to which he gave liberally of his time and money. He was one of the chief promoters of the Columbian Dental Congress. He also was for many years an active worker in the National Dental Association, and the National Board of Dental Examiners. It was largely through his efforts that the first state reciprocity action was taken. He was one of our giants and it seem hard to realize that he has gone from us, but his works and a grateful memory remains to all who had the pleasure of knowing him.

